# Active surveillance of highly suspicious thyroid nodules cohort in China shows a worse psychological status in younger patients

**DOI:** 10.3389/fonc.2022.981495

**Published:** 2022-08-26

**Authors:** Chunhao Liu, Hao Zhao, Yu Xia, Yue Cao, Liyang Zhang, Ya Zhao, Luying Gao, Ruifeng Liu, Yuewu Liu, Hongfeng Liu, Zhilan Meng, Shuzhou Liu, Xiaoyi Li

**Affiliations:** ^1^ Department of General Surgery, Peking Union Medical College Hospital, Chinese Academy of Medical Sciences & Peking Union Medical College, Beijing, China; ^2^ Department of Ultrasound, Peking Union Medical College Hospital, Chinese Academy of Medical Sciences & Peking Union Medical College, Beijing, China; ^3^ Department of Pathology, Peking Union Medical College Hospital, Chinese Academy of Medical Sciences & Peking Union Medical College, Beijing, China; ^4^ Department of Otolaryngology Head and Neck Surgery, Hainan General Hospital, Haikou, China

**Keywords:** highly suspicious thyroid nodules, active surveillance, management, outcome, psychological status

## Abstract

**Background:**

Active surveillance has been considered a safe alternative to surgery for low-risk papillary thyroid microcarcinoma. This study aimed to assess the oncological outcomes and psychological status of active surveillance of highly suspicious thyroid nodules ≤10 mm in China.

**Methods:**

This prospective single-center cohort study enrolled 336 patients with highly suspicious thyroid nodules for active surveillance to assess oncological outcomes and psychological status. The psychological status of patients was assessed by two different questionnaires and compared among different patient groups.

**Results:**

During a median follow-up period of 28.5 months, eight patients underwent delayed surgery for tumor enlargement and one for lymph node metastases. The cumulative incidence of disease progression at 5 and 10 years was 6.0% and 12.8%, respectively. Patients who underwent delayed surgery had no permanent complications, and no patient had distant metastasis or death. Patients ≤30 years old had a higher baseline anxiety score (4.9 vs. 3.8, p=0.024), a higher proportion of baseline anxiety score, i.e., ≥8 points (24.0% vs. 12.6%, p=0.033), and a worse baseline emotional function (62.7 vs. 70.7, p=0.013) than patients >30. During AS, patients ≤30 years of age had higher overall anxiety levels (p=0.005) and a worse overall emotional function (p=0.001).

**Conclusions:**

Active surveillance in Chinese patients with highly suspicious subcentimetre thyroid nodules has good oncological outcomes and can be used as a safe alternative to surgery. Younger patients (≤30) show a worse psychological status; therefore, more attention should be paid to younger patients.

## Introduction

The worldwide incidence of thyroid cancer has significantly increased over the past three decades (1, 2), and more than 50% of this increase is linked to the identification of papillary thyroid microcarcinoma (PTMC) (3, 4). As shown in several autopsy studies, if never diagnosed and treated, most PTMCs remain stable without influencing overall survival (5, 6). Two prospective studies from Japan have shown that AS is feasible for low-risk PTMC patients, given that most of these tumors remain latent without progression or with very slow progression (7, 8). Other studies have also confirmed the feasibility and safety of AS (9–14). In addition, the prognosis of PTMC was excellent, with a local or regional recurrence rate of 2–6%, a distant metastasis rate of 1–2%, and a 20-year disease-specific survival rate of >99% (15, 16). For most low-risk PTMC patients, immediate surgery may lead to more problems, such as surgical complications, neck scarring, and hormone replacement therapy (17, 18). Therefore, AS can be used as an alternative treatment option to reduce the incidence of adverse events without affecting treatment outcomes (19). The incidence of thyroid cancer has also increased rapidly in China, with around 200,000 new cases in 2015, similar to the global trend (20). In our institution, approximately 70% of thyroid surgery patients are diagnosed with PTMC, and most of these tumors are low-risk PTMCs (21). However, there are no prospective studies reporting AS in patients with low-risk PTMC in China.

Psychological status is important for many diseases, including cancer, and it is especially relevant for chronic diseases, which can significantly influence treatment decisions and outcomes (22). Low-risk PTMC resembles a chronic disease; hence, in addition to its oncological treatment, an assessment of psychological status is also important. Few studies have evaluated the psychological status in low-risk PTMC patients, suggesting that surgical patients experience more psychological problems (23, 24). Therefore, it is valuable to determine how the psychological status of low-risk PTMC patients changes in China during AS and how it interacts with treatment decisions and outcomes.

Fine-needle aspiration biopsy (FNAB) is considered the most accurate and cost-effective method available for evaluating thyroid nodules, recommended for nodules ≥10 mm in the greatest dimension with a high-suspicion sonographic pattern. For highly suspicious nodules ≤10 mm, FNAB could be postponed until therapy is considered (11, 25). However, the rates of non-diagnostic and indeterminate cytological results in thyroid nodules ≤10 mm can reach up to 35.6% and 60%, respectively (26–28). In addition, FNAB is not generally well accepted in China; the evaluation and diagnosis of thyroid nodules ≤10 mm is usually conducted by ultrasound. The 2015 ATA ultrasound malignancy risk stratification of thyroid nodules has a good diagnostic performance and plays a key role in the identification and management of thyroid nodules (29). We used these criteria to reach a “thyroid microcarcinoma” diagnosis of highly suspicious nodules, explore AS outcomes for these highly suspicious nodule patients without high-risk factors, and determine its interaction with psychological status in China.

## Materials and methods

### Study design and patients

This prospective cohort study included 336 patients diagnosed with highly suspicious thyroid nodules by ultrasound and followed up by AS without immediate surgery from 2018 to 2021 at Peking Union Medical College Hospital (PUMCH), Beijing, China. We included highly suspicious nodules ≤10 mm assessed by the 2015 ATA ultrasound malignancy risk stratification of thyroid nodules without the following high-risk factors: a) extrathyroidal invasion or clinical lymph node metastasis (LNM) and b) nodules adjacent to recurrent laryngeal nerve, trachea, or esophagus (11). Exclusion criteria were: a) a history of previous thyroid cancer surgery, b) patients refusing to sign informed consent, c) patients who could not adhere to regular follow-ups, d) a family history of thyroid cancer (≥2 family members diagnosed with thyroid cancer) and e) a history of previous mental illness. The following parameters were recorded in this study: gender, age at diagnosis, follow-up time, educational level, marital status, economic level, past medical history, family history, hormone replacement therapy, ultrasound features, the results of the FNAB if performed, surgical approach, postoperative pathology, surgical complications, follow-up outcomes, laboratory tests (TSH, Tg, Tg-Ab, Tpo-Ab, CEA), chest low-dose computed tomography (CT), and two psychological status questionnaires: the Hospital Anxiety and Depression Scale (HADS) Questionnaire and the European Organization for Research and Treatment of Cancer (EORTC) Quality of Life Core Questionnaire (EORTC QLQ-C30) (30). Neck Ultrasound was performed using a Philips iU 22 machines with a 5–12-MHz transducer by two radiologists (X.Y, G.L.Y). This study was approved by the Ethics Review Committee of PUMCH (Ethics number: JS-2454), and each patient signed an informed consent form and was informed that the lesion was clinically diagnosed as a malignancy.

### Follow-up plan

**Figure 1:**
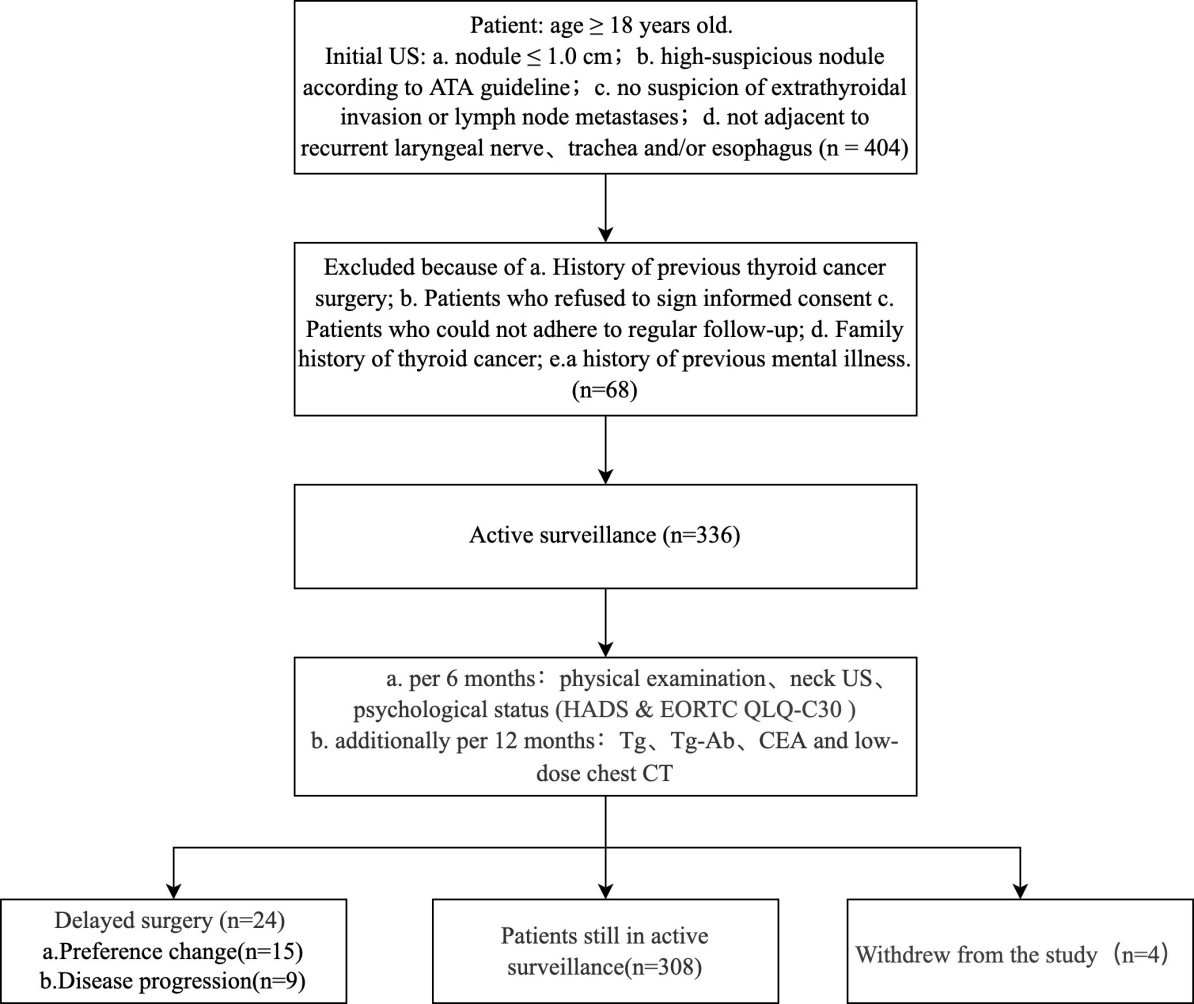
Flowchart of Active Surveillance.

Patients were regularly followed up with physical examinations and neck ultrasounds every six months. Psychological status questionnaires were also collected every six months. In addition, serum Tg, Tg-Ab, CEA, and chest low-dose CT examinations were performed every 12 months (**Figure 1**). Delayed surgery was performed when the patient showed the following conditions: a) a tumor enlargement defined by a size increase of 3 mm or more compared with the size at the initiation of observation and subsequent FNAB confirming malignancy, b) novel LNM or distant metastasis, c) invasion of recurrent laryngeal nerve, trachea or esophagus, and d) changes in the preference of the patient. Patients’ surgical approach and postoperative follow-up plan were implemented in accordance with Chinese guidelines (31). The primary endpoints in this study included the disease progression rate and the patient’s psychological status. The secondary endpoints included the preference change rate and tumor persistence/recurrence rate.

### Psychological status questionnaires

The Hospital Anxiety and Depression Scale (HADS) Questionnaire consists of fourteen items. Seven of them indicated anxiety, and the remaining seven indicated depression. The scores of the anxiety and depression subscale were divided into 0–7, no problems of clinical relevance (non-cases); 8–10, cases that warrant further psychiatric investigation (possible cases); and ≥11, clinical level of anxiety/depression (probable cases) (32).

The European Organization for Research and Treatment of Cancer (EORTC) Quality of Life Core Questionnaire (EORTC QLQ-C30) consists of five functional scales (physical, role, emotional, social, and cognitive), three symptom scales (fatigue, pain, and nausea/vomiting), a global health status scale, and six single-item scales (dyspnea, loss of appetite, insomnia, constipation, diarrhea, and financial difficulties) (30).

### Statistical analyses

Statistical analyses were performed using SPSS (IBM, Version 26.0), GraphPad Prism (Version 9.1.1), and R studio (Version 4.1.0). Categorical variables were expressed as numbers and percentages; continuous variables were expressed as mean ± standard deviation (SD), or median (range). A standard chi-squared test was used to compare categorical variables. The t-test or the Wilcoxon’s test were used to compare continuous variables. A competing risk model was used to analyze the cumulative incidence of disease progression and preference change and associated risk factors. Mixed linear models were used to analyze validated psychological scale results and associated risk factor analysis. All p-values were two-sided, and p<0.05 was considered statistically significant.

## Results

### Baseline clinical features

Clinical features of the 336 patients under active surveillance (AS) are listed in **Table 1**. The median age was 43 years old (range: 19–75). In total, 264 patients (78.6%) were female. According to age at diagnosis, 187 patients (55.7%) were ≤45 years old, and 149 (44.3%) were >45 years old. The mean maximal tumor diameter at initial diagnosis was 0.58 ± 0.19 cm, and 184 patients (54.8%) had tumors >0.5 cm in diameter. Multifocality was found in 82 patients (24.7%). The mean serum TSH concentration was 2.05 mIU/L; three patients (0.9%) received levothyroxine replacement therapy, and four (1.2%) had a family history of thyroid cancer (non-medullary thyroid cancer, and only one family member was diagnosed). Thirteen patients (3.9%) had a history of other malignancies. FNAB was performed in 62 patients, and the percentage of malignancy and suspicion for malignancy was 87.1%. CEA was positive in two cases (0.7%), one case had undergone surgery and was confirmed to be PTC, and one case was tested with normal calcitonin levels.

**Table 1:** Baseline clinical features of patients under active surveillance.

Item	Value (N = 336)
Gender	
Male	72 (21.4%)
Female	264 (78.6%)
Age	
Mean ± SD	43.7 ± 11.7
Median (Range)	43 (19~75)
≤45 yrs	187 (55.7%)
>45 yrs	149 (44.3%)
≤30 yrs	50 (14.9%)
>30 yrs	286 (85.1%)
Follow-up time (months)	
Mean ± SD	32.9 ± 23.4
Median (Range)	28.5 (4.3~138)
Family history of thyroid cancer	
Yes	4 (1.2%)
No	332 (98.8%)
Hormone replacement therapy	
Yes	3 (0.9%)
No	333 (99.1%)
Tumor diameter ^a^	0.58 ± 0.19
≤ 0.5cm	152 (45.2%)
> 0.5cm	184 (54.8%)
Multifocality	
Yes	82 (24.7%)
No	254 (75.3%)
FNAB	62 (18.5%)
Malignant	46 (74.2%)
Suspicious for malignancy	8 (12.9%)
AUS/FLUS	3 (4.8%)
Nondiagnostic or unsatisfactory	5 (8.1%)
Laboratory test	
TSH (0.380-4.340μIU/ml)	2.05 ± 1.08
Negative	324 (96.1%)
Positive	13 (3.9%)
Tg-Ab (< 115 IU/ml)	
Positive	53 (15.8%)
Negative	283 (84.2%)
TPO-Ab (< 34 IU/ml)	307 (91.4%)
Positive	45 (14.7%)
Negative	262 (85.3%)
Tg (1.4-78.0 ng/ml)	325 (96.7%)
Positive	8 (2.5%)
Negative	317 (97.5%)
CEA (≤5ng/ml)	1.55 ± 1.01
Positive	2 (0.7%)
Negative	307 (99.3%)

a Diameter of the largest lesion in multifocal tumors. SD, standard deviation; yrs, years old; FNAB, Fine-needle aspiration biopsy; AUS/FLUS, atypia of undetermined significance/follicular lesion of undetermined significance; TSH, thyroid stimulating hormone; Tg-Ab, thyroglobulin antibody; CEA, carcinoma embryonic antigen.

### Outcome of patients with delayed surgery

During a median follow-up of 28.5 months (range: 4.3–138), 24 patients (7.1%) underwent delayed surgery. The median age of the patients was 41 years old (range: 25–66), and 87.5% were female. Four patients (1.2%) withdrew from the study. The most common reason for delayed surgery was a change in patient’s preference (15 cases), followed by disease progression (eight cases of tumor enlargement and one case of novel LNM). Of the 24 patients who underwent delayed surgery, 17 underwent lobectomy with central neck dissection, and seven patients underwent total thyroidectomy with central neck dissection. Postoperative pathology showed 15 cases of classic PTC, nine cases of follicular variant PTC, eight cases of LNM, and two cases of high-volume LNM. Only three patients developed transient hypocalcemia after surgery, and no other complications occurred. During a median postoperative follow-up of 7.3 months (range: 0.2–23.3), one patient was found to have lateral neck LNM after one year of follow-up, underwent lateral cervical lymph node dissection (LNM ratio: 1/28), and then reached disease-free survival. No distant metastasis or death occurred. There were no significant differences in gender, capsular invasion, pathological subtype, multifocality, and LNM between the preference change group and disease progression group (**Supplementary Table S1**).

**Figure 2:**
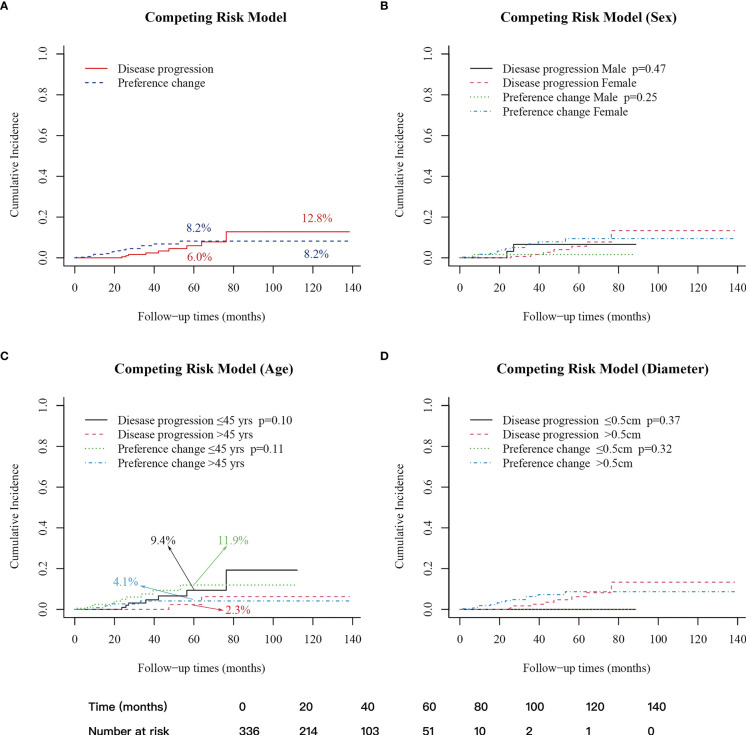
Cumulative incidence of patients with disease progression and preference change. **(A)** 5-year cumulative incidence: disease progression (6.0%); preference change (8.2%); 10-year cumulative incidence: disease progression (12.8%); preference change (8.2%). **(B)** Between male and female groups. **(C)** Between age>45 years old and age ≤45 years old groups, 5-year cumulative incidence: disease progression ≤ 45 years old (9.4%); disease progression>45 years old (2.3%); preference change ≤ 45 years old (11.9%); preference change>45 years old (4.1%). **(D)** Between diameter ≤ 0.5cm and>0.5cm. A p-value < 0.05 was considered significant.

A competing risk model analysis showed that the 5- and 10-year cumulative incidence of patients with disease progression were 6.0% and 12.8%, respectively, and the 5- and 10-year cumulative incidence of patients with preference change were 8.2% and 8.2%, respectively (**Figure 2A**). Regarding gender, age (45 years old as the boundary) and tumor size (0.5 cm as the boundary), the cumulative incidences were not significantly different in either the disease progression group or the preference change group (**Figures 2B–D**).

### Baseline questionnaire score and risk factor analysis

The mean Hospital Anxiety and Depression Scale A (HADS-A) score was 4.0 ± 3.3 (range: 0–15), and there were 47 (14.3%) cases with a HADS-A score of ≥8. The mean Hospital Anxiety and Depression Scale D (HADS-D) score was 3.8 ± 3.1 (range: 0–15), and there were 42 (12.5%) cases with a HADS-D score of ≥8 (**Tables 2**, **3**). Compared with patients still under AS, the HADS-A score (4.0 ± 3.2 vs. 4.2 ± 3.2, p=0.794) and HADS-D score (3.9 ± 3.1 vs. 3.8 ± 3.1, p=0.924) of patients who changed their preference to surgery were not statistically different (**Table 4**). The proportion of HADS-A score ≥8 (13.3% vs. 14.4%, p>0.999) and HADS-D score ≥8 (20.0% vs. 12.1%, p=0.415) were also not statistically different according to preference change or not (**Table 3**). Patients ≤30 years of age had a higher HADS-A score than patients >30 (4.9 ± 3.3 vs. 3.8 ± 3.2, p=0.024) (**Table 4**) and represented a higher proportion of HADS-A score of ≥8 (24.0% vs. 12.6%, p=0.033) (**Table 3**). The baseline score of each item from the EORTC QLQ-C30 was not significantly different in terms of preference change. Patients ≤30 years of age had a worse emotional function than those >30 (62.7 ± 20.4 vs. 70.7 ± 21.1, p=0.013) (**Table 5**).

**Table 2:** Baseline score of HADS and EORTC QLQ-C30.

HADS baseline score							
Item	Mean ± SD	Mode	Minimum	Percentile 25	Percentile 50	Percentile 75	Maximum
HADS-T	7.8 ± 5.8	2.0	0.0	3.0	7.0	11.0	30.0
HADS-A	4.0 ± 3.3	3.0	0.0	1.0	3.0	6.0	15.0
HADS-D	3.8 ± 3.1	1.0	0.0	1.0	3.0	6.0	15.0
EORTC QLQ-C30 baseline score							
Item	Mean ± SD	Mode	Minimum	Percentile 25	Percentile 50	Percentile 75	Maximum
Physical function	86.9 ± 13.1	100.0	20.0	80.0	93.3	100.0	100.0
Role function	91.5 ± 16.6	100.0	0.0	83.3	100.0	100.0	100.0
Emotional function	69.5 ± 21.1	66.7	0.0	58.3	66.7	83.3	100.0
Cognitive function	79.5 ± 20.1	83.3	0.0	66.7	83.3	100.0	100.0
Social function	92.7 ± 14.2	100.0	33.3	83.3	100.0	100.0	100.0
Global Health status	76.9 ± 20.4	83.3	0.0	66.7	83.3	91.7	100.0
Fatigue	31.3 ± 21.7	33.3	0.0	22.2	33.3	44.4	100.0
Nausea & vomiting	8.0 ± 12.8	0.0	0.0	0.0	0.0	16.7	66.7
Pain	14.2 ± 16.3	0.0	0.0	0.0	16.7	16.7	100.0
Dyspnea	21.0 ± 25.3	0.0	0.0	0.0	0.0	33.3	100.0
Insomnia	38.6 ± 33.9	33.3	0.0	0.0	33.3	66.7	100.0
Appetite loss	13.7 ± 21.2	0.0	0.0	0.0	0.0	33.3	100.0
Constipation	23.3 ± 26.6	0.0	0.0	0.0	33.3	33.3	100.0
Diarrhea	21.7 ± 24.9	0.0	0.0	0.0	33.3	33.3	100.0
Financial difficulties	7.0 ± 17.1	0.0	0.0	0.0	0.0	0.0	100.0

HADS-T, HADS Total score; HADS-A, HADS Anxiety score; HADS-D, HADS Depression score.

**Table 3:** Univariate analysis of risk factors for HADS baseline scores.

Item	Sex			Age			Preference change		
	Male (N=72)	Female (N=264)	P-value	≤30 yrs (N=50)	>30 yrs (N=286)	P-value	No (N=321)	Yes (N=15)	P-value
HADS-T									
<15	62 (86.1%)	227 (86.0%)	0.978	41 (82.0%)	248 (86.7%)	0.375	276 (86.0%)	13 (86.7%)	>0.999
≥15	10 (13.9%)	37 (14.0%)		9 (18.0%)	38 (13.3%)		45 (14.0%)	2 (13.3%)	
HADS-A									
<8	62 (86.1%)	226 (85.6%)	0.914	38 (76.0%)	250 (87.4%)	0.033	275 (85.7%)	13 (86.7%)	>0.999
≥8	10 (13.9%)	38 (14.4%)		12 (24.0%)	36 (12.6%)		46 (14.3%)	2 (13.3%)	
HADS-D									
<8	62 (86.1%)	232 (87.9%)	0.688	46 (92.0%)	248 (86.7%)	0.362	282 (87.9%)	12 (80.0%)	0.414
≥8	10 (13.9%)	32 (12.1%)		4 (8.0%)	38 (13.3%)		39 (12.1%)	3 (20.0%)	

HADS-T, HADS Total score; HADS-A, HADS Anxiety score; HADS-D, HADS Depression score; yrs, years old

**Table 4:** HADS mean baseline scores of patients under AS, depending on Sex, Age and Treatment options.

Item	Sex (Mean ± SD)			Age (Mean ± SD)			Preference change (Mean ± SD)		
	Male (N=72)	Female (N=264)	P-value	≤30 yrs (N=50)	>30 yrs (N=286)	P-value	No (N=321)	Yes (N=15)	P-value
HADS-T	7.5 ± 5.5	7.9 ± 5.9	0.651	8.8 ± 5.9	7.6 ± 5.8	0.180	7.8 ± 5.8	8.1 ± 6.4	0.844
HADS-A	4.0 ± 3.1	4.0 ± 3.3	0.933	4.9 ± 3.3	3.8 ± 3.2	0.024	4.0 ± 3.2	4.2 ± 3.2	0.794
HADS-D	3.5 ± 3.1	3.9 ± 3.1	0.348	3.9 ± 3.1	3.8 ± 3.1	0.867	3.8 ± 3.1	3.9 ± 3.5	0.924

HADS-T, HADS Total score; HADS-A, HADS Anxiety score; HADS-D, HADS Depression score; SD: standard deviation;yrs: years old.

**Table 5:** EORTC QLQ-C30 baseline mean scores of patients under AS, depending on Sex, Age and Treatment options.

Item	Sex (Mean ± SD)			Age (Mean ± SD)			Preference change (Mean ± SD)		
	Male (N=72)	Female (N=264)	P-value	≤30 yrs (N=50)	>30 yrs (N=286)	P-value	No (N=321)	Yes (N=15)	P-value
**Physical function**	90.9 ± 11.7	85.8 ± 13.3	0.002	86.5 ± 13.8	87.0 ± 13.0	0.817	86.8 ± 13.0	87.1 ± 14.6	0.954
**Role function**	93.5 ± 16.4	91.0 ± 16.6	0.249	93.0 ± 18.2	91.3 ± 16.3	0.495	91.4 ± 16.6	91.1 ± 18.8	0.922
**Emotional function**	72.8 ± 20.0	68.6 ± 21.4	0.135	62.7 ± 20.4	70.7 ± 21.1	0.013	69.4 ± 21.4	66.1 ± 18.8	0.528
**Cognitive function**	82.9 ± 18.1	78.5 ± 20.6	0.105	82.0 ± 18.1	79.0 ± 20.4	0.334	79.2 ± 20.3	78.9 ± 18.3	0.911
**Social function**	93.7 ± 11.7	92.4 ± 14.8	0.483	91.7 ± 14.4	92.9 ± 14.2	0.575	92.9 ± 13.9	87.8 ± 19.4	0.169
**Global Health status**	78.0 ± 19.7	76.5 ± 20.7	0.592	76.2 ± 15.7	77.0 ± 21.2	0.795	76.5 ± 20.8	80.0 ± 15.7	0.543
**Fatigue**	26.2 ± 21.0	32.6 ± 21.7	0.025	32.9 ± 20.9	31.0 ± 21.8	0.572	31.1 ± 21.7	36.3 ± 23.9	0.359
**Nausea & vomiting**	5.8 ± 10.9	8.7 ± 13.2	0.093	13.7 ± 16.0	7.1 ± 11.9	0.007	8.0 ± 12.8	8.9 ± 12.4	0.792
**Pain**	8.8 ± 13.4	15.7 ± 16.7	0.001	12.7 ± 13.7	14.5 ± 16.7	0.476	14.3 ± 16.4	14.4 ± 16.5	0.951
**Dyspnea**	11.6 ± 21.0	23.6 ± 25.7	<0.001	24.7 ± 29.2	20.4 ± 24.5	0.271	20.6 ± 25.2	31.1 ± 29.5	0.114
**Insomnia**	32.4 ± 33.6	40.3 ± 33.9	0.081	30.7 ± 30.7	40.0 ± 34.3	0.073	38.1 ± 33.3	48.9 ± 43.4	0.358
**Appetite loss**	11.1 ± 19.4	14.4 ± 21.6	0.216	12.0 ± 18.8	14.0 ± 21.6	0.541	13.1 ± 20.2	24.4 ± 36.7	0.257
**Constipation**	15.7 ± 23.0	25.4 ± 27.1	0.006	20.7 ± 25.1	23.8 ± 26.8	0.446	23.6 ± 26.9	26.7 ± 22.5	0.618
**Diarrhea**	24.5 ± 24.4	20.9 ± 25.0	0.280	24.0 ± 21.3	21.3 ± 25.4	0.432	21.8 ± 24.8	26.7 ± 28.7	0.432
**Financial difficulties**	4.6 ± 12.9	7.7 ± 18.0	0.104	9.3 ± 17.9	6.6 ± 16.9	0.326	7.5 ± 17.6	2.2 ± 8.6	0.050

AS, active surveillance; SD, standard deviation; yrs, years old.

### Mixed linear model analysis of HADS and EORTC QLQ-C30

**Figure 3:**
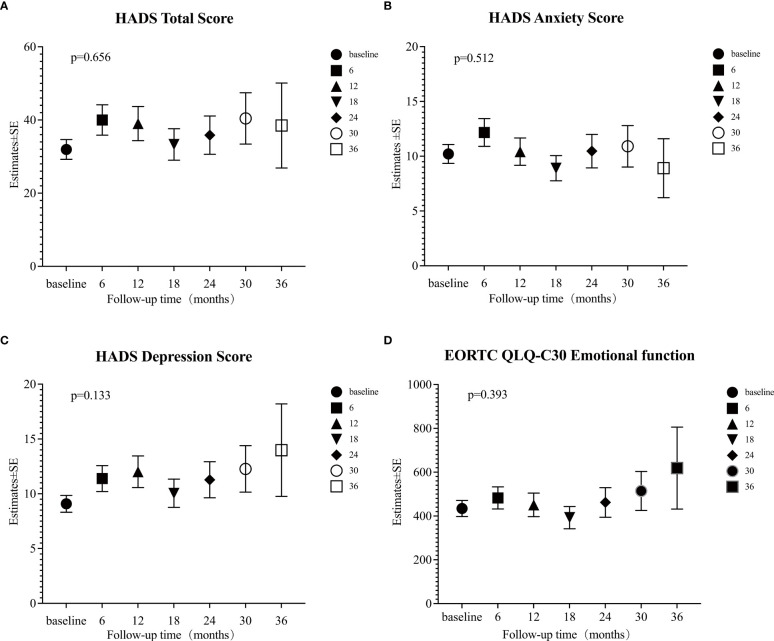
Estimates of four psychological scales. **(A)** Hospital Anxiety and Depression Scale (HADS) total score; **(B)** HADS anxiety score; **(C)** HADS depression score; **(D)** European Organization for Research and Treatment of Cancer (EORTC) Quality of Life Core Questionnaire (EORTC QLQ-C30) Emotional function was calculated at each time point (baseline, and 6, 12, 18, 24, 30, and 36 months) by mixed linear model. A p-value < 0.05 was considered significant.

**Figure 4:**
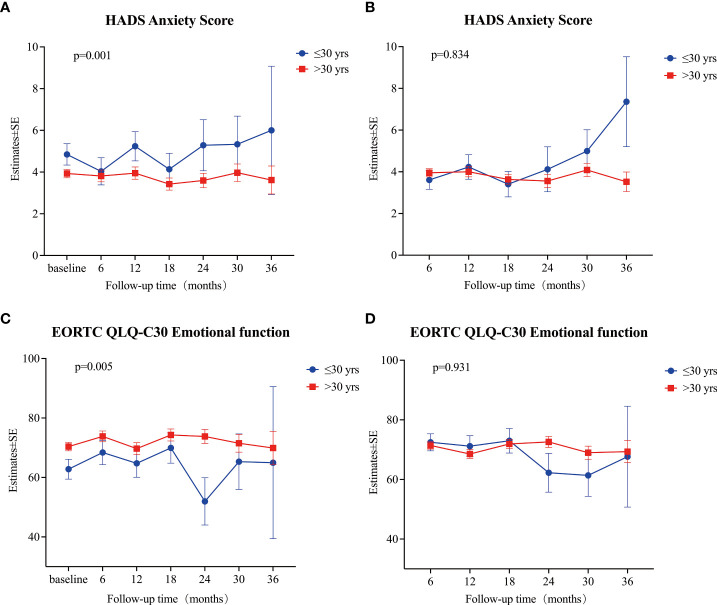
**(A)** Estimates of HADS anxiety score at each time point (baseline, and 6, 12, 18, 24, 30, and 36 months) between patients ≤30 years old and patients > 30 years old. **(B)** Estimates of HADS anxiety score at each time point (6, 12, 18, 24, 30, and 36 months) between patients ≤30 years old and patients > 30 years old after adjusting baseline score. **(C)** Estimates of EORTC QLQ-C30 Emotional function at each time point (baseline, and 6, 12, 18, 24, 30, and 36 months) between patients ≤30 years old and patients > 30 years old. **(D)** Estimates of EORTC QLQ-C30 Emotional function at each time point (6, 12, 18, 24, 30, and 36 months) between patients ≤30 years old and patients > 30 years old after adjusting baseline score. All estimates were calculated using a mixed linear model. A p-value < 0.05 was considered significant.

The mixed linear model analysis of HADS showed no significant differences in the HADS-T, HADS-A, and HADS-D at different follow-up times (all p>0.05; **Supplementary Table S2**, **Figures 3A-C**). Risk factor analysis showed that during the follow-up, the overall mean anxiety score in patients aged ≤30 was higher than that of patients >30 (Estimate=0.89, 95% CI: 0.27~1.52, p=0.005) (**Supplementary Table S2**, **Figure 4A**). After adjusting the baseline HADS-A score as a covariate, there were no significant differences between the overall mean anxiety score of patients ≤30 years old and that of patients >30 (Estimate=0.064, 95% CI: -0.54~0.67, p=0.834) (**Supplementary Table S4**, **Figure 4B**). There were no significant differences in the overall mean scores of HADS-T, HADS-A, and HADS-D between male and female groups (**Supplementary Table S2).**

The mixed linear model analysis of EORTC QLQ-C30 showed no significant differences at different follow-up times, except for nausea/vomiting and financial difficulties (**Supplementary Table S3**, **Figure 3D**). Risk factor analysis showed that men had better scores than women in physical, role, cognitive, and social functions, fatigue, pain, dyspnea, insomnia, constipation, and financial difficulties (**Supplementary Table S3**). The overall mean score of emotional function was lower in patients ≤30 years old than in those >30 (Estimate = 6.90, 95% CI: 2.82–10.98, p = 0.001) (**Supplementary Table S3**, **Figure 4C**). After adjusting baseline emotional function as a covariate, the overall mean of the emotional function of patients ≤30 years old was not significantly different from that of patients >30 (Estimate= -0.168, 95% CI: -3.99~3.66, p =0.931) (**Supplementary Table S4**, **Figure 4D**).

## Discussion

The global incidence of papillary thyroid cancer has substantially increased over the past few decades, with more than half of this increase linked to PTMC (33, 34). Researchers from Japan firstly proposed AS as an alternative strategy to immediate surgery for low-risk PTMC patients (35). Subsequently, several other research teams also successively reported studies on AS of low-risk PTMC (36–38). Our study is the first prospective active surveillance cohort study in China reporting oncological outcomes and psychological burden of low-risk thyroid microcarcinomas diagnosed clinically according to the 2015 ATA ultrasound malignancy risk stratification of thyroid nodules (11). As the acceptance of FNAB in China is less favorable, in this study, only 62 patients (18.5%) underwent FNAB. A low rate of FNAB may lead to misdiagnosis, but ultrasound still has relatively high diagnostic sensitivity (about 95%) and accuracy (about 85%) values for malignant nodules (29, 39). A total of 336 patients were included in this study. The median follow-up time was 28.5 months. Nine patients (2.7%) showed disease progression: eight patients (2.4%) showed tumor enlargement, and one patient (0.3%) showed newly identified LNM. The cumulative incidence of disease progression at five years was 6.0%, similar to the incidence reported (4.4–6.4%) in a systematic review (40). Delayed surgery was performed in 24 patients. The primary reason for delayed surgery was a change in patient’s preference (15/24), followed by tumor enlargement (8/24) and LNM (1/24), which are similar to previous studies (12, 36). The overall LNM rate of patients with delayed surgery of 33.3% was comparable to the LNM rate from previous studies (41). Patients with disease progression had no worse postoperative pathological outcomes compared to patients who changed their preference, and none of the patients had permanent surgery-related complications. During a median postoperative follow-up of 7.3 months (0.2-23.3), only one patient was found to have lateral neck LNM, and an excellent outcome was achieved after the second surgery. The preliminary outcome of this study showed that AS is safe and feasible for patients with highly suspicious thyroid nodules in China

Several studies have suggested that PTMC patients who undergo immediate surgery experience more health-related problems, including mental health issues, than patients who are managed by AS (23, 24), which may influence patients’ treatment decisions and outcomes. The main reason for delayed surgery was a change in preference rather than disease progression in previous studies and this study (12, 36). Although we did not find significant differences in baseline anxiety score (HADS) and emotional function (EORTC QLQ-C30) between patients who changed their preference for surgery and those who did not, we showed that patients ≤30 years of age had a significantly higher baseline anxiety score (4.9 ± 3.3 vs. 3.8 ± 3.2, p=0.024), a significantly worse baseline emotional function (62.7 ± 20.4 vs. 70.7 ± 21.1, p=0.013), and a significantly higher baseline proportion of patients with a HADS-A score of ≥8 (24.0% vs. 12.6%, p=0.033) than patients >30 (**Tables 3**, **4**, and **Supplementary Table S2**). Among patients with a preference change, 13.3% of patients were younger than or equal to 30 years old, and 15.0% for patients still under AS, the similar percentage might explain no differences in scores. On the other hand, this study also showed that during follow-ups, patients ≤30 years of age had a higher anxiety score (**Supplementary Table S2**, **Figure 4A**) and a worse emotional function (**Supplementary Table S3**, **Figure 4C**). After adjusting the effect of the baseline score, we observed that for patients ≤30 years of age, a lower overall psychological status was mainly related to a worse baseline psychological status. For the first time, we found that younger patients had a worse baseline psychological status, which led to a worse psychological status during the follow-up period. Therefore, psychological counseling might be necessary for patients with highly suspicious thyroid nodules managed by AS, and it might improve the psychological status during the follow-up period, especially in younger patients.

Age is an important prognostic factor of differentiated thyroid cancer (11, 41, 42). Previous studies have found that PTC in young patients may be more progressive than in older patients during AS (7, 36, 43). This study compared the cumulative incidence of disease progression across different age stratifications, which showed no significant difference. However, the 5-year cumulative incidence of disease progression between patients aged ≤45 and patients >45 was significantly different (9.4% vs. 2.3%, p=0.1) (**Figure 2C**). Also, among patients who changed their preference, the 5-year cumulative incidence was higher in patients aged ≤45 compared with patients >45 (11.9% vs. 4.1%, p=0.11) (**Figure 2C**). These findings show no significant differences, which may be related to the small number of patients and the shorter follow-up time. According to previous studies and the data from this study, the disease progression still requires special attention for younger patients, especially for those with a longer life expectancy and a longer follow-up time. Moreover, these patients may be more likely to change their preference, requiring attention not only to oncological outcomes but also to psychological status assessment.

This study had certain limitations, such as the malignancy criteria of highly suspicious thyroid nodules (they have mainly been diagnosed by ultrasound, which may have low misdiagnosis rates), the relatively short follow-up period, and a small number of cases (it may be necessary to continue to expand the sample size and increase the follow-up time to obtain more robust data). In addition, most patients do not have their initial visit to our institution. Therefore, there may be some bias when conducting the psychological questionnaire assessment.

In conclusion, active surveillance of highly suspicious subcentimetre thyroid nodules in patients without high-risk factors has good oncological outcomes and could be a safe alternative to surgery in China. However, younger patients may not only be more prone to disease progression but also have a worse psychological status, which may affect their treatment process; therefore, more attention should be paid to younger patients.

## Data availability statement

The raw data supporting the conclusions of this article will be made available by the authors, without undue reservation.

## Author contributions

CL: Conceptualization, methodology, validation, formal analysis, data curation, visualization, writing - original draft. HZ: Investigation, formal analysis, data curation. YX: Conceptualization, and, methodology, writing - review and editing. YC: Investigation, Data Curation. LZ: Investigation, Data Curation. YZ: Investigation, Data Curation. LG: Investigation, Data Curation. RL: Investigation, Data Curation. YL: Conceptualization, methodology, writing - review and editing. HL: investigation, data curation. ZM: Methodology. SL: Investigation, Data Curation. XL: Conceptualization, investigation, methodology, Project administration, supervision, Funding acquisition, writing-review and editing. All authors contributed to the article and approved the submitted version.

## Funding

This work was supported by the Non-profit Central Research Institute Fund of Chinese Academy of Medical Sciences (grant numbers: 2019XK320011).

## Acknowledgments

The authors thank Mr. Yanlong Li for statistical guidance.

## Conflict of interest

The authors declare that the research was conducted in the absence of any commercial or financial relationships that could be construed as a potential conflict of interest.

## Publisher’s note

All claims expressed in this article are solely those of the authors and do not necessarily represent those of their affiliated organizations, or those of the publisher, the editors and the reviewers. Any product that may be evaluated in this article, or claim that may be made by its manufacturer, is not guaranteed or endorsed by the publisher.
