# Supplementary material for: Active surveillance of highly suspicious thyroid nodules cohort in China shows a worse psychological status in younger patients
Source: Front Oncol. 2022 Aug 26;12:981495. doi: 10.3389/fonc.2022.981495 (PMC9458970; doi:10.3389/fonc.2022.981495)
Supplement: Supplementary file 1 [file Table_1.docx]

Supplementary Table S1. Clinicopathological data of patients with delayed surgery

| Item | Preference change | Disease progression | P-value |
| --- | --- | --- | --- |
| Gender |  |  |  |
| Male | 1 （6.7%） | 2 (22.2%) | 0.533 |
| Female | 14（93.3%） | 7 (77.8%) |  |
| Age |  |  |  |
| Mean ± SD | 43.2±11.8 | 40.7±10.1 |  |
| Median (Range) | 44 (24-66) | 39 (26-60) |  |
| ≤45 yrs | 11（73.3%） | 7（77.8%） | >0.999 |
| ＞45 yrs | 4 （26.7%） | 2（22.2%） |  |
| ≤30 yrs | 2（13.3%） | 1（11.1%） | >0.999 |
| ＞30 yrs | 13（86.7%） | 8（88.9%） |  |
| TSH（0.380-4.340μIU/ml） | 1.74±0.67 | 1.71±0.69 | 0.503 |
| Tumor diameter ^a^ | 0.59±0.16 | 0.65±0.22 | >0.999 |
| ≤ 0.5cm | 4 （26.7%） | 3（33.3%） |  |
| >0.5cm | 11（73.3%） | 6（66.7%） |  |
| Operation |  |  |  |
| TOTAL+CLND | 4 （25.0%） | 3（33.3%） |  |
| LOB+CLND | 11（68.7%） | 6（66.7%） |  |
| Pathological subtype |  |  | 0.080 |
| Classic | 7 （46.7%） | 8（88.9%） |  |
| Follicular variant | 8 （53.3%） | 1（11.1%） |  |
| Capsule invasion |  |  | 0.625 |
| Yes | 5 （33.3%） | 4（44.4%） |  |
| No | 10（66.7%） | 5（55.6%） |  |
| Multifocality |  |  | 0.668 |
| Yes | 5 （33.3%） | 3（33.3%） |  |
| No | 10（66.7%） | 6（66.7%） |  |
| LNM |  |  | 0.325 |
| No | 11（73.3%） | 5（55.6%） |  |
| Yes | 4 （26.7%） | 4（44.4%） |  |
| ≤ 5 metastatic LN | 3 | 3 |  |
| ＞5 metastatic LN | 1 | 1 |  |
| Postoperative complications |  |  |  |
| Temporary hypocalcemia | 2 | 1 |  |
| Persistent/Recurrence |  |  |  |
| LNM | 1 | 0 |  |

^a^ Diameter of the largest lesion in multifocal tumors; TOTAL+CLND: Total thyroidectomy + central lymph node dissection; LOB+CLND: Lobectomy + central lymph node dissection; LNM: lymph node metastasis; LN: lymph node
