# Supplementary material for: Active surveillance of highly suspicious thyroid nodules cohort in China shows a worse psychological status in younger patients
Source: Front Oncol. 2022 Aug 26;12:981495. doi: 10.3389/fonc.2022.981495 (PMC9458970; doi:10.3389/fonc.2022.981495)
Supplement: Supplementary file 2 [file Table_2.docx]

Supplementary Table S2. Mixed linear model analysis of HADS score

| Parameter | Estimate | P-value | 95% Confidence Interval | |
| --- | --- | --- | --- | --- |
|  |  |  | Lower Bound | Upper Bound |
| HADS-T |  |  |  |  |
| Intercept | 7.87 | <0.001 | 7.25 | 8.49 |
| Follow-up times | 0.01 | 0.656 | -0.03 | 0.05 |
| Male | -0.14 | 0.777 | -1.12 | 0.84 |
| Female | 0^a^ | . | . | . |
| ≤30 yrs | 0.68 | 0.254 | -0.49 | 1.84 |
| >30 yrs | 0^a^ | . | . | . |
| HADS-A |  |  |  |  |
| Intercept | 3.84 | <0.001 | 3.50 | 4.19 |
| Follow-up times | -0.01 | 0.512 | -0.03 | 0.01 |
| Male | 0.13 | 0.633 | -0.40 | 0.66 |
| Female | 0^a^ | . | . | . |
| ≤30 yrs | 0.89 | 0.005 | 0.27 | 1.52 |
| >30 yrs | 0^a^ | . | . | . |
| HADS-D |  |  |  |  |
| Intercept | 4.02 | <0.001 | 3.69 | 4.35 |
| Follow-up times | 0.02 | 0.133 | 0.00 | 0.04 |
| Male | -0.26 | 0.329 | -0.80 | 0.27 |
| Female | 0^a^ | . | . | . |
| ≤30 yrs | -0.22 | 0.500 | -0.84 | 0.41 |
| >30 yrs | 0^a^ | . | . | . |

^a^ This parameter is set to zero because it is redundant.

HADS-T= HADS Total score; HADS-A= HADS Anxiety score; HADS-D= HADS Depression score；yrs: years old
