# Supplementary material for: Active surveillance of highly suspicious thyroid nodules cohort in China shows a worse psychological status in younger patients
Source: Front Oncol. 2022 Aug 26;12:981495. doi: 10.3389/fonc.2022.981495 (PMC9458970; doi:10.3389/fonc.2022.981495)
Supplement: Supplementary file 3 [file Table_3.docx]

Supplementary Table S3. Mixed linear model analysis of EORTC QLQ-C30

| Parameter | Estimate | P-value | 95% Confidence Interval | |
| --- | --- | --- | --- | --- |
|  |  |  | Lower Bound | Upper Bound |
| **Physical function** |  |  |  |  |
| Intercept | 85.82 | <0.001 | 84.40 | 87.24 |
| Follow-up times | -0.06 | 0.197 | -0.14 | 0.03 |
| Male | 6.31 | 0.000 | 4.02 | 8.60 |
| Female | 0^a^ | . | . | . |
| ≤30 yrs | -0.76 | 0.583 | -3.49 | 1.96 |
| >30 yrs | 0^a^ | . | . | . |
| **Role function** |  |  |  |  |
| Intercept | 90.55 | <0.001 | 88.74 | 92.36 |
| Follow-up times | -0.09 | 0.120 | -0.19 | 0.02 |
| Male | 3.37 | 0.022 | 0.49 | 6.25 |
| Female | 0^a^ | . | . | . |
| ≤30 yrs | 1.21 | 0.489 | -2.22 | 4.63 |
| >30 yrs | 0^a^ | . | . | . |
| **Cognitive function** |  |  |  |  |
| Intercept | 76.37 | <0.001 | 74.18 | 78.56 |
| Follow-up times | -0.04 | 0.502 | -0.17 | 0.08 |
| Male | 5.40 | 0.002 | 1.96 | 8.83 |
| Female | 0^a^ | . | . | . |
| ≤30 yrs | 3.25 | 0.120 | -0.85 | 7.35 |
| >30 yrs | 0^a^ | . | . | . |
| **Emotional function** |  |  |  |  |
| Intercept | 69.57 | <0.001 | 67.35 | 71.80 |
| Follow-up times | 0.06 | 0.393 | -0.08 | 0.20 |
| Male | 3.36 | 0.057 | -0.10 | 6.82 |
| Female | 0^a^ | . | . | . |
| ≤30 yrs | -6.90 | 0.001 | -10.98 | -2.82 |
| >30 yrs | 0^a^ | . | . | . |
| **Social function** |  |  |  |  |
| Intercept | 92.19 | <0.001 | 90.60 | 93.78 |
| Follow-up times | 0.06 | 0.142 | -0.02 | 0.15 |
| Male | 2.62 | 0.039 | 0.14 | 5.09 |
| Female | 0^a^ | . | . | . |
| ≤30 yrs | -2.19 | 0.150 | -5.17 | 0.79 |
| >30 yrs | 0^a^ | . | . | . |
| **Global Health status** |  |  |  |  |
| Intercept | 76.70 | <0.001 | 74.62 | 78.79 |
| Follow-up times | 0.02 | 0.788 | -0.10 | 0.13 |
| Male | 3.20 | 0.046 | 0.05 | 6.35 |
| Female | 0^a^ | . | . | . |
| ≤30 yrs | -3.21 | 0.094 | -6.97 | 0.54 |
| >30 yrs | 0^a^ | . | . | . |
| Fatigue |  |  |  |  |
| Intercept | 32.87 | <0.001 | 30.58 | 35.15 |
| Follow-up times | -0.03 | 0.601 | -0.16 | 0.09 |
| Male | -7.39 | <0.001 | -10.96 | -3.82 |
| Female | 0^a^ | . | . | . |
| ≤30 yrs | 3.18 | 0.144 | -1.09 | 7.44 |
| >30 yrs | 0^a^ | . | . | . |
| **Nausea & vomiting** |  |  |  |  |
| Intercept | 7.09 | <0.001 | 5.81 | 8.37 |
| Follow-up times | -0.10 | 0.006 | -0.17 | -0.03 |
| Male | -0.76 | 0.452 | -2.74 | 1.22 |
| Female | 0^a^ | . | . | . |
| ≤30 yrs | 5.56 | <0.001 | 3.17 | 7.94 |
| >30 yrs | 0^a^ | . | . | . |
| Pain |  |  |  |  |
| Intercept | 16.20 | <0.001 | 14.46 | 17.94 |
| Follow-up times | -0.03 | 0.558 | -0.13 | 0.07 |
| Male | -5.74 | <0.001 | -8.47 | -3.00 |
| Female | 0^a^ | . | . | . |
| ≤30 yrs | -2.00 | 0.226 | -5.23 | 1.24 |
| >30 yrs | 0^a^ | . | . | . |
| **Dyspnea** |  |  |  |  |
| Intercept | 23.35 | <0.001 | 20.75 | 25.95 |
| Follow-up times | -0.02 | 0.742 | -0.17 | 0.12 |
| Male | -9.41 | <0.001 | -13.39 | -5.43 |
| Female | 0^a^ | . | . | . |
| ≤30 yrs | 0.80 | 0.739 | -3.93 | 5.54 |
| >30 yrs | 0^a^ | . | . | . |
| Insomnia |  |  |  |  |
| Intercept | 41.22 | <0.001 | 37.75 | 44.69 |
| Follow-up times | 0.05 | 0.613 | -0.16 | 0.27 |
| Male | -6.49 | 0.017 | -11.84 | -1.15 |
| Female | 0^a^ | . | . | . |
| ≤30 yrs | -8.74 | 0.007 | -15.06 | -2.41 |
| >30 yrs | 0^a^ | . | . | . |
| **Appetite loss** |  |  |  |  |
| Intercept | 15.24 | <0.001 | 13.00 | 17.48 |
| Follow-up times | 0.00 | 0.959 | -0.13 | 0.12 |
| Male | -2.44 | 0.165 | -5.89 | 1.01 |
| Female | 0^a^ | . | . | . |
| ≤30 yrs | -3.49 | 0.096 | -7.60 | 0.63 |
| >30 yrs | 0^a^ | . | . | . |
| Constipation |  |  |  |  |
| Intercept | 25.46 | <0.001 | 22.65 | 28.27 |
| Follow-up times | -0.02 | 0.807 | -0.19 | 0.15 |
| Male | -7.92 | <0.001 | -12.24 | -3.60 |
| Female | 0^a^ | . | . | . |
| ≤30 yrs | -1.30 | 0.618 | -6.39 | 3.80 |
| >30 yrs | 0^a^ | . | . | . |
| **Diarrhea** |  |  |  |  |
| Intercept | 22.44 | <0.001 | 19.71 | 25.17 |
| Follow-up times | -0.05 | 0.525 | -0.22 | 0.11 |
| Male | 2.82 | 0.186 | -1.36 | 7.00 |
| Female | 0^a^ | . | . | . |
| ≤30 yrs | -0.04 | 0.986 | -4.98 | 4.89 |
| >30 yrs | 0^a^ | . | . | . |
| **Financial difficulties** |  |  |  |  |
| Intercept | 7.74 | <0.001 | 5.99 | 9.48 |
| Follow-up times | -0.13 | 0.002 | -0.21 | -0.05 |
| Male | -2.87 | 0.027 | -5.41 | -0.33 |
| Female | 0^a^ | . | . | . |
| ≤30 yrs | 2.31 | 0.141 | -0.77 | 5.39 |
| >30 yrs | 0^a^ | . | . | . |

^a^ This parameter is set to zero because it is redundant. yrs: years old
