# Supplementary material for: Active surveillance of highly suspicious thyroid nodules cohort in China shows a worse psychological status in younger patients
Source: Front Oncol. 2022 Aug 26;12:981495. doi: 10.3389/fonc.2022.981495 (PMC9458970; doi:10.3389/fonc.2022.981495)
Supplement: Supplementary file 4 [file Table_4.docx]

Supplementary Table S4. Mixed linear model analysis of HADS-A and EORTC QLQ-C30 Emotional function after adjusting baseline score

| Parameter | Estimate | P-value | 95% Confidence Interval | |
| --- | --- | --- | --- | --- |
|  |  |  | Lower Bound | Upper Bound |
| HADS-A |  |  |  |  |
| Intercept | 1.12 | <0.001 | 0.62 | 1.61 |
| Follow-up times | 0.00 | 0.808 | -0.02 | 0.02 |
| Male | 0.26 | 0.304 | -0.24 | 0.77 |
| Female | 0^a^ |  |  |  |
| ≤30 yrs | 0.06 | 0.834 | -0.54 | 0.67 |
| >30 yrs | 0^a^ |  |  |  |
| Baseline score | 0.71 | <0.001 | 0.64 | 0.77 |
| Emotional function |  |  |  |  |
| Intercept | 23.48 | <0.001 | 18.37 | 28.60 |
| Follow-up times | -0.05 | 0.507 | -0.19 | 0.10 |
| Male | -0.08 | 0.962 | -3.28 | 3.13 |
| Female | 0^a^ |  | . | . |
| ≤30 yrs | -0.17 | 0.931 | -3.99 | 3.66 |
| >30 yrs | 0^a^ |  | . | . |
| Baseline score | 0.71 | <0.001 | 0.64 | 0.77 |

^a^ This parameter is set to zero because it is redundant. HADS-A= HADS Anxiety score；yrs: years old
